# Protective Effects of Resveratrol on TNF-*α*-Induced Endothelial Cytotoxicity in Baboon Femoral Arterial Endothelial Cells

**DOI:** 10.1155/2013/185172

**Published:** 2013-03-31

**Authors:** Juan Xiao, Jun Song, Vida Hodara, Allen Ford, Xing Li Wang, Qiang Shi, Li Chen, John L. VandeBerg

**Affiliations:** ^1^Department of Endocrinology, Qilu Hospital, Shandong University, 107 Wen Hua Xi Lu, Jinan, Shandong 250012, China; ^2^Southwest National Primate Research Center, Texas Biomedical Research Institute, P.O. Box 760549, San Antonio, TX 78245-0549, USA; ^3^Cardiothoracic Research Laboratory, Texas Heart Institute, Baylor College of Medicine, Houston, TX 77030-2604, USA

## Abstract

Endothelial injury induced by inflammatory factors plays a critical role in the pathogenesis of cardiovascular disease. Endothelial cell (EC) apoptosis, proliferation, migration, and cellular adhesion molecule (CAM) expression contribute to the development of atherosclerosis. We investigated the effects of resveratrol (0.1–100 **μ**M) on the proliferation, migration, and CAM expression of primary cultures of baboon arterial endothelial cells (BAECs). In addition, we tested its effects under normal conditions as well as under inflammatory conditions induced by tumour necrosis factor-*α* (TNF-*α*) administered either by cotreatment, pretreatment, or posttreatment. Immunocytochemistry, MTT, wound-healing, and flow cytometry assays were performed. The resveratrol treatment significantly enhanced BAEC proliferation and attenuated TNF-*α*-induced impairment of proliferation at the optimal doses of 1–50 *µ*M. Resveratrol at a high dose (100 **μ**M) and TNF-*α* impaired BAEC migration, while low doses of resveratrol (1–50 **μ**M) attenuated TNF-*α*-induced impairment of BAEC migration. Moreover, resveratrol inhibited TNF-*α*-induced ICAM-1 and VCAM-1 expression. Taken together, our results suggest that the resveratrol protects BAECs after inflammatory stimulation as well as ameliorates inflammatory effects at low concentrations. Consequently, resveratrol should be considered as a candidate drug for the prevention and treatment of inflammatory vascular diseases.

## 1. Introduction

Endothelial cell (EC) cytotoxicity induced by inflammatory factors plays a key role in the pathogenesis of cardiovascular disease. Tumour necrosis factor (TNF)-*α*, a pleiotropic proinflammatory cytokine involved in the pathogenesis of inflammatory, and vascular disease, can promote endothelial cell apoptosis and inflammation [1] by directly activating a number of cellular stress-sensitive pathways including nuclear factor-kappa B (NF-*κ*B) and mitogen-activated protein kinase (MAPK) [2]. These subsequently contribute to endothelial cell injury and cellular dysfunction [3]. Therefore, the inhibition of TNF-*α*-induced endothelial cell cytotoxicity can be important in preventing the cardiovascular disease and inhibiting its progression. 

The resveratrol possesses many pharmacological properties including anticancer [4], anti-inflammation [5], and cardioprotective effects [6]. The resveratrol exerts direct cardiovascular protective effects by improving myocardial perfusion, reducing oxidant stress, and inhibiting platelet aggregation [7–9]. Recent studies have shown that resveratrol acts partially through the inhibition of cellular apoptosis and inflammation by inhibiting the NF-*κ*B pathway [10, 11]. The beneficial effects of resveratrol on suggest that it could be an important pharmacological target for the treatment of cardiovascular disease. However, resveratrol has cell-specific [12, 13] and dose-dependent [14] effects on cellular proliferation or apoptosis. Studies of a wide range of concentrations of resveratrol administered to a uniform population of ECs are lacking. Such studies are required to comprehend the diverse and sometimes contradictory cellular effects of resveratrol [15]. Previous studies demonstrated that resveratrol has biphasic properties in relation to its concentration on EC proliferation, with no effects at low concentrations (0.1–25 *μ*M) and induced apoptosis at high doses (such as 100 *μ*M) in human umbilical vein endothelial cells (HUVECs) [16]. 

The baboon is a well-characterized model for human biomedical studies including cardiovascular diseases [17, 18]. In the present study, we investigated the effects of a wide range of concentrations of resveratrol on cultured primary baboon arterial endothelial cells (BAECs) under normal conditions as well as under TNF-*α*-induced inflammatory condition in which cells underwent cotreatment, pretreatment, or posttreatment with resveratrol. 

## 2. Methods

### 2.1. Materials

Unless otherwise indicated, all the reagents used in this study and their sources were as follows: resveratrol and 3-(4,5-dimethyl-2-thiazoyl)-2,5-diphenyl-2H-tetrazolium bromide (MTT) (Sigma, St. Louis, MO); anti-CD62E (E-selectin) (R&D Systems, Minneapolis, MN, Clone BB1G-E5); anti-CD54 (intercellular adhesion molecule-1) (BD Biosciences, San Jose, CA, Clone HA58); anti-CD106 (vascular cell adhesion molecule-1) (US Biological, Swampscott, MA, Clone 5K26T); cell culture media and supplies (Invitrogen, Carlsbad, CA).

### 2.2. BAEC Isolation and Culture

BAECs were isolated from baboon femoral arteries as previously described [17]. Briefly, a 2-3 cm segment of femoral artery was obtained under sterile surgical procedure by experienced veterinarians; procedures were approved by the Institutional Animal Care and Use Committee of Texas Biomedical Research Institute. The artery was incubated with 0.1% collagenase at 37°C for 15 min to digest extracellular tissue to allow the release of cells. The released cells were washed and seeded immediately on 1.0% gelatin-coated culture plates. Primary BAECs were cultured in F-12K growth medium supplemented with 20% foetal calf serum (FCS). Passages three and four of BAECs were used in this study. BAECs were treated with resveratrol at various concentrations in the presence or absence of TNF-*α* (10 ng/mL) for different time periods. 

### 2.3. Immunocytochemistry

Primary BAECs were seeded into Lab-Tek multiwell chamber slides (BD Falcon 8-well Culture Slides) [18], fixed with 200 *μ*L 4% formaldehyde at room temperature (RT) for 10 min and then blocked with 10% goat serum for 30 min at RT. Cells were then incubated with 300 *μ*L of 1 : 400 or 1 : 800 diluted primary antibody (vWF: Sigma, St. Louis, MO; VE-Cadherin: Cell Signaling Technology, Danvers, MA) in 1% BSA in phosphate buffered saline (PBS) overnight at 4°C and then with FITC-labelled secondary antibody in 1% BSA in PBS for 1 h at RT in the dark. DAPI (0.5 *μ*g/mL) was added for 5 min, and slides were mounted with 10 *μ*L/well antifade solution (Invitrogen, no. S36936) in 1× PBS. Slides were observed by fluorescence microscopy.

### 2.4. MTT Assay

Cells were seeded in 96-well plates at a density of 1000 cells/well in 2% FCS F-12K growth medium for 24 h and treated with resveratrol at the designated concentrations (0.1–100 *μ*mol/L) in the presence or absence of TNF-*α* (10 ng/mL) for different time periods. Treated cells were incubated with 20 *μ*L (1 mg/mL) MTT for 4 h at 37°C to form formazan crystals by reacting with metabolically active cells. Subsequently, the formazan crystals were solubilized with 150 *μ*L DMSO. The absorbance of each well was measured at A570 nm using a microplate reader. Cell viability was measured by the absorbance, normalized to cell numbers, incubated in control medium (considered 100%), and then determined relative to the control.

### 2.5. Wound-Healing Assay

Nearly confluent cell monolayers were “wounded” in a cross-shaped pattern with a sterile 200 *μ*L pipette tip. The medium and dislodged cells were aspirated, and plates were replenished by either endothelial cell growth medium (ECGM) without FCS, which served as the control or by medium containing resveratrol (0.1–100 *μ*M) with or without TNF-*α* (10 ng/mL). Images of the wound healing at 15 h were captured in the same scratched area localized to the right of the cross-shaped scratch with a 40x objective (Olympus IX70 microscope) and quantified by measuring the wound area with Image-Pro Plus software 6.0. Three fields per well were evaluated, and all experiments were performed in quadruplicate. Results were reported as the percentage of wound healing using the equation: % wound healing = [1 − (wound area at T15 h /wound area at T0 h)] × 100, where T0 h is the time immediately following wounding. 

### 2.6. Flow Cytometry

The expression of proportions of cell specific cellular adhesion molecules was quantified by standard immunofluorescence cell sorting techniques [19, 20]. The following antibodies were used in this study: anti-human CD62E (clone BBIG-E5), anti-human CD54 (clone HA58), and anti-human CD106 (clone 5K267). Unstained and isotype controls were used to determine background staining. Flow cytometry analysis was performed using Cyan ADP (Becton Dickinson, San Jose, CA). Mean fluorescence intensity (MFI) values in respective gates were used to represent antigen expression. All experiments were performed three times and each time in triplicate.

### 2.7. Statistical Analysis

All quantitative variables were expressed as means ± SEM from at least three separate experiments. Comparisons between groups were made using one-way ANOVA followed by Dunnett's post hoc test. To evaluate the difference of two groups, we used two-tailed Student's *t*-test. *P* < 0.05 was considered statistically significant. SPSS version 17.0 (SPSS Inc., Chicago, IL) was used for statistical analyses. 

## 3. Results

### 3.1. Primary BAEC Culture and Identification

**Figure 1:**
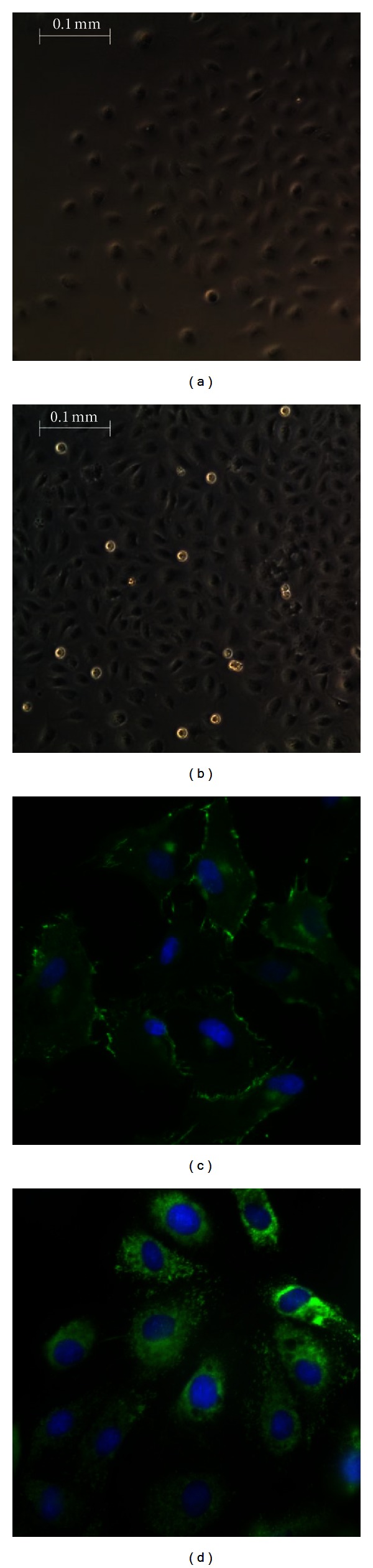
Morphology of primary baboon ECs cultured at day 3 (a) and day 7 (b). Isolated baboon femoral artery ECs showed a typical cobble stone shape (magnification ×40). Cells were stained with specific endothelial cell surface markers for VE-cadherin (c) and vWF (d) (magnification ×100). Immunofluorescence staining of baboon ECs with antibody to VE-cadherin and von Willebrand factor was conducted using an FITC-labelled secondary antibody. DAPI was used to stain the nuclei. Images were taken with an Eclipse E800 microscope.

Primary baboon arterial had a typical cobblestone shape (Figures 1(a) and 1(b)) and took 3–7 days to reach confluence depending on cell seeding density and number of passages. BAECs maintained proliferation ability for 30 populations or 10 passages, with stable expression of cellular adhesion molecules. Prior to their use, we stained BAECs with endothelial specific marker VE-cadherin (Figure 1(c)) and vWF (Figure 1(d)) to ensure the purity and healthy condition of the cells.

### 3.2. Resveratrol Enhances BAEC Proliferation and Attenuates TNF-*α*-Induced Impairment

**Figure 2:**
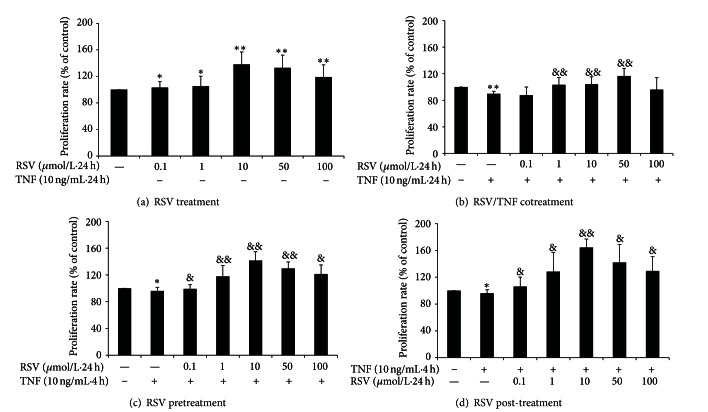
Effect of resveratrol on BAEC proliferation with or without TNF-*α* for 24 h. Resveratrol (0.1–100 *μ*M) highly significantly enhanced EC proliferation after 24 h incubation in the optimal range of 10–50 *μ*M (a). TNF-*α* (10 ng/mL) inhibited endothelial cell proliferation, while cotreatment with resveratrol (1–50 *μ*M) reversed inhibition by TNF-*α* (b). BAECs were pretreated with resveratrol for 24 h then treated with TNF-*α* for 4 h; pretreatment with resveratrol ameliorated TNF-*α*-induced inhibition (c). BAECs were treated with TNF-*α* for 4 h then incubated with resveratrol for 24 h; posttreatment with resveratrol ameliorated TNF-*α*-induced inhibition (d). Data are expressed as a percentage of basal value (100%) and are the mean ± SEM from seven independent experiments, each conducted in quintuplicate. *P < 0.05, **P < 0.01 versus control and ^&^ *P* < 0.05; and ^&&^ *P* < 0.01 versus TNF-*α* alone.

We first tested the effect of various concentrations of resveratrol (0.1–100 *μ*M) on the growth of primary BAECs (Figure 2(a)) and the extent to which resveratrol affected BAEC proliferation impaired by TNF-*α* when administered as cotreatment (Figure 2(b)), pretreatment (Figure 2(c)), or posttreatment (Figure 2(d)). Resveratrol (0.1–100 *μ*M) highly significantly enhanced EC proliferation after 24 h incubation in the optimal range of 10–50 *μ*M (*P* < 0.05, *P* < 0.01) (Figure 2(a)). TNF-*α* treatment alone (10 ng/mL) for 24 h significantly decreased (*P* < 0.01) BAEC proliferation by comparison with controls (Figure 2(b)). However, cotreatment with resveratrol at 1–50 *μ*M alleviated cytotoxicity induced by TNF-*α* (*P* < 0.01). No effects were detected at 1 or 100 *μ*M, suggesting a dose-dependent effect of resveratrol on baboon ECs subjected to inflammatory conditions. Pretreatment with resveratrol (0.1–100 *μ*M) (Figure 2(c)) for 24 h also attenuated impairment of BAEC proliferation caused by incubation with TNF-*α* for 4 h (*P* < 0.05), especially at doses of 1–50 *μ*M (*P* < 0.01). Additionally, posttreatment with resveratrol (0.1–100 *μ*M) (Figure 2(d)) for 24 h attenuated TNF-*α*-induced impairment of BAEC proliferation, with the optimal dose being 10 *μ*M (*P* < 0.05, *P* < 0.01). Together, the data indicated that the resveratrol could enhance primary baboon EC proliferation and could prevent cytotoxicity induced by TNF-*α*.

### 3.3. Resveratrol Attenuated BAEC Migration Impaired by TNF-*α*

**Figure 3:**
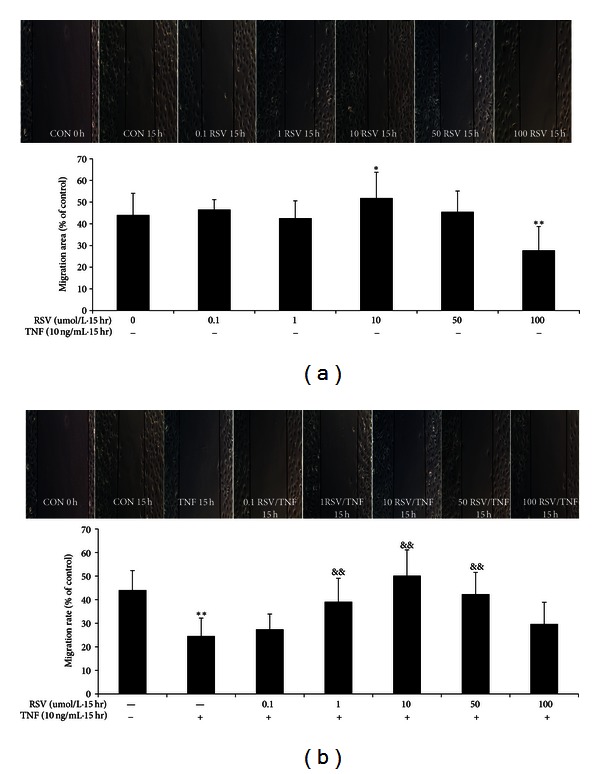
Effect of resveratrol on baboon endothelial cell migration with or without TNF-*α* (10 ng/mL) for 15 h. 10 *μ*M resveratrol significantly increased BAEC migration, and 100 *μ*M resveratrol significantly decreased BAEC migration (a). TNF-*α* administration (b) decreased BAEC migration by comparison with controls. Resveratrol (1–50 *μ*M) attenuated impairment of BAEC migration by TNF-*α* when during incubation for 15 h (b). Data are the mean ± SEM from five independent experiments; each conducted in triplicate. *P < 0.05; and **P < 0.01 versus control, ^&^ *P* < 0.05; and ^&&^ *P* < 0.01 versus TNF-*α* alone.

We investigated whether resveratrol could affect BAEC migration with or without TNF-*α* treatment. As shown in Figure 3(a), resveratrol at a low concentration (10 *μ*M; *P* < 0.05) increased wounded BAEC migration by comparison with controls, but, at a high concentration (100 *μ*M; *P* < 0.01), it decreased the BAEC migration during 15 h. No effect was observed at concentrations of 0.1, 1, and 50 *μ*M. As shown in Figure 3(b), TNF-*α* (*P* < 0.01) dramatically impaired BAEC migration at 15 h compared with controls, while resveratrol (1–50 *μ*M) in the presence of TNF-*α* significantly increased the BAEC migration rate by comparison with TNF-*α* treatment alone (*P* < 0.01). No protective effect of 100 *μ*M resveratrol was observed.

### 3.4. Resveratrol Inhibits TNF-*α*-Induced Expression of VCAM-1 (CD106) and ICAM-1 (CD54) in BAECs

**Figure 4:**
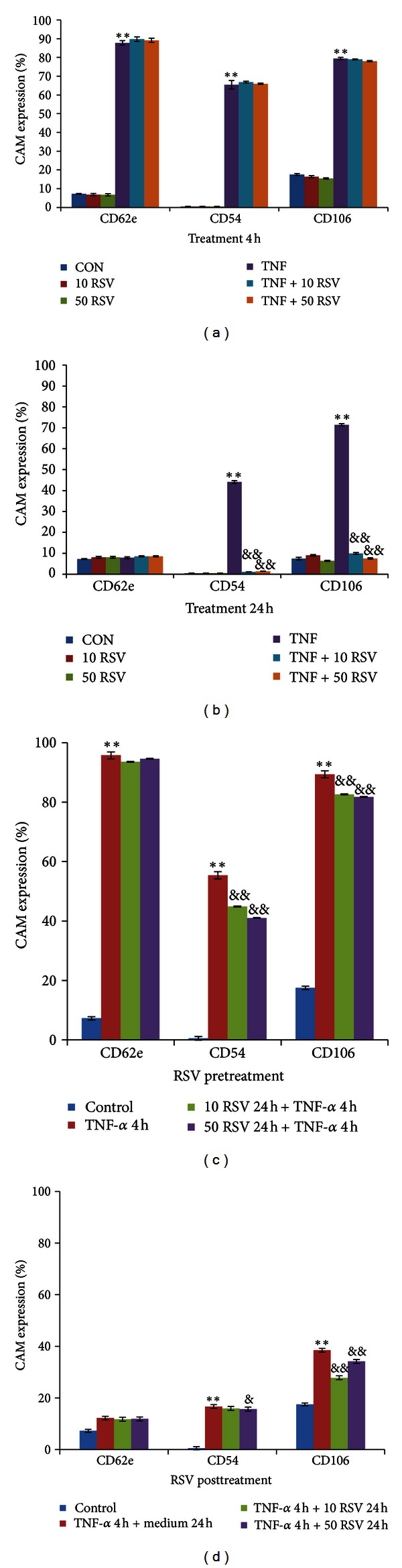
Resveratrol inhibited CAM expression induced by TNF-*α* (10 ng/mL). TNF-*α* significantly stimulated ICAM (CD54) and VCAM (CD106) expression after 4 H (a) and 24 h (b) treatment and significantly increased E-selectin (CD62e) expression after 4 h. Resveratrol (RSV) (10, 50 *μ*M) inhibited expression of ICAM and VCAM in activated BAECs stimulated with TNF-*α* (10 ng/mL) during 24 h of cotreatment (b). Resveratrol (10, 50 *μ*M) administered pretreatment (c) also significantly inhibited the expression of ICAM and VCAM activated by TNF-*α*, and significantly attenuated sustained ICAM and VCAM expression after TNF stimulation by posttreatment (d). Data are mean ± SEM from three independent experiments each performed in triplicate. *P < 0.05; and **P < 0.01 versus control and ^&^ *P* < 0.05; and ^&&^ *P* < 0.01 versus TNF-*α* alone.

To investigate whether resveratrol affected the level of inducible cell adhesion molecule expression of E-selectin (CD62E), ICAM-1 (CD54), and VCAM-1 (CD106), BAECs were analysed by flow cytometry. BAECs were cotreated (Figures 4(a) and 4(b)), pretreated (Figure 4(c)), or posttreated (Figure 4(d)) with resveratrol (10, 50 *μ*M) and TNF-*α*. E-selectin, ICAM-1, and VCAM-1 expression on BAECs were significantly increased by TNF-*α* (10 ng/mL) stimulation at 4 h by comparison with controls (*P* < 0.01, Figure 4(a)). ICAM-1 and VCAM-1, but not E-selectin, remained elevated after 24 h (*P* < 0.01, Figure 4(b)). Resveratrol cotreatment did not inhibit the elevation in CAM expression elicited by TNF-*α* at 4 h (Figure 4(a)), but the expression of ICAM-1 and VCAM-1 significantly decreased in BAECs treated for 24 h with resveratrol (10, 50 *μ*M) and TNF-*α* (10 ng/mL) by comparison with TNF-*α* treatment alone (10 ng/mL, *P* < 0.01, Figure 4(b)). Pretreatment with resveratrol (10, 50 *μ*M) for 24 h (Figure 4(c)) inhibited the elevation of ICAM-1 and VCAM-1 expression induced by TNF-*α* stimulation (*P* < 0.01). BAECs that were treated with TNF-*α* for 4 h and then incubated with resveratrol (10, 50 *μ*M, posttreatment) for 24 h (Figure 4(d)) exhibited significantly attenuated ICAM-1 (50 *μ*M) and VCAM-1 (10, 50 *μ*M) expression compared with controls (*P* < 0.05, *P* < 0.01).

## 4. Discussion

Recently, resveratrol has gained considerable attention because of its anticancer [4], anti-inflammatory [5], antidiabetes [21], and cardiovascular protective effects [6]. These properties have been reported in studies using experimental mouse, rat, and swine models [8, 22], but not primate models. Moreover, the effects of resveratrol on human are influenced by their vascular origin [23]. 

In this study, we investigated the effect of resveratrol on primary baboon femoral artery as an *in vitro* primate model. The advantages of this model are as follows. First, it is easier to obtain primary macrovascular ECs from baboons in comparison with humans. Second, baboons exhibit a high degree of genetic, biochemical, physiological, and anatomical similarity to humans, and more closely resemble humans in comparison with other species in the pathology of vascular diseases [18]. Third, we preferred BAECs because they are hyperresponsive to TNF-*α* compared with venous ECs, and they may play a key role in macrovascular disease under adverse conditions [24]. Fourth, BAECs have similar responses to human aortic endothelial cells (HAECs) when stimulated with TNF-*α* and thus provide an effective model for translating therapies to human clinical trials [25]. BAECs can be used at an early stage of disease that may closely mimic the inflammation responses of ECs *in vivo*. We have established a protocol for primary BAEC culture and previously performed fundamental investigations on their morphology and functions [20, 26]. Additionally, Mikula-Pietrasik et al. recently demonstrated that the resveratrol and its derivatives have cell-specific effects, which were confirmed by having different effects on the proliferation of human from different sources [15]. Thus, we selected BAECs as our model for *in vitro* studies as a prelude to *in vivo* studies.

Proliferation and migration of ECs may play a crucial role in vascular self-repair in normal physiological as well as pathological conditions. EC monolayer integrity is maintained via proliferation and migration of neighboring cells. We first investigated different experimental settings to verify the effects of resveratrol on BAEC proliferation under normal and inflammatory conditions. Resveratrol highly significantly enhanced BAEC proliferation at the optimal concentrations of 10–100 *μ*M, and it attenuated cellular impairment caused by TNF-*α* treatment when administered to the cells as cotreatment, pretreatment, or post-treatment. Data concerning the influence of resveratrol on EC growth have not been completely consistent. Previous studies demonstrated that resveratrol induces increasing levels of apoptosis in with increasing concentration (25–100 *μ*M) and induces apoptosis at 100 *μ*M by cleavage of caspase 3 [16, 27], while low doses (0.1–25 *μ*M) had no effect. Csiszar et al. demonstrated that 10 *μ*M resveratrol prevented EC apoptosis induced by cigarette smoke extract [28]. Our results were partially consistent with those studies. Moreover, Ungvari et al. and Brito et al. showed that resveratrol at a concentration range of 1–100 *μ*M attenuated apoptosis mediated by oxidative stress (TNF-*α*, oxidized LDL, and peroxynitrite) [29, 30]. With respect to EC apoptosis, the impact of resveratrol depends on concentration as well as cell source. Our study suggested that resveratrol is beneficial in protecting BAECs subjected to inflammatory stimulation as well as preventing cell injury at low concentrations. 

The translation of *in vitro* and *in vivo* findings from experiments of animals and humans is thought to depend largely on parent resveratrol plasma concentration and bioavailability. The greatest plasma concentrations of resveratrol have been reported to be 2.36 *μ*M by Boocock et al. [31] and 4.2 *μ*M by Brown et al. [32] when 5 g of transresveratrol was administered to humans. However, the physiological plasma levels are either not detectable or below the micromolar concentrations that are typically employed *in vitro* (~32 nM–100 *μ*M) in research with human cells. However, tissue resveratrol levels may be higher than what is suggested based on plasma levels because resveratrol is lipophilic. Moreover, resveratrol has shown efficacy at very low concentrations [33] in studies with animal models of human diseases and dramatically opposite effects depending on dose [34]. Our present studies investigated the protective effects of low concentrations of resveratrol on baboon ECs. Additional experiments with baboons are needed to determine if the *in vitro* effects of resveratrol on inflammation reflect *in vivo* effects that can be achieved by oral ingestion of resveratrol and if oral ingestion is cardioprotective. 

EC migration is an essential process for a variety of vascular functions such as tumour growth, vascular remodelling, and vascular wound healing. Our results indicated that resveratrol at a low concentration ameliorates impairment of BAEC migration induced by TNF-*α* (0.1–50 *μ*M). We found that a low concentration of resveratrol had no inhibitory effect on BAEC migration. However, a high concentration (100 *μ*M) of resveratrol decreased BAEC migration. In et al. [27] showed that resveratrol inhibited endothelial cell migration at a dose range of 10–100 *μ*M in human and bovine brain EC because of its antiangiogenesis properties. Our results also demonstrated that low doses of resveratrol attenuated the impairment of BAEC migration caused by inflammatory conditions, suggesting a mechanism for its anti-inflammatory properties. Cicha et al. [35] observed that resveratrol dose-dependently inhibited EC migration at 1–20 *μ*mol/L in a Rho-associated-kinase- (ROCK-) dependent manner. Resveratrol promoted proliferation and migration of cerebral by activation of phosphoinositide 3 kinase (PI3-K)/Akt and mitogen-activated protein kinase (MAPK)/ERK signaling pathways [36]. However, a recent study also revealed that polyphenols from olive oil and red wine reduce inflammatory EC migration in cultured through MMP-9 and COX-2 inhibition [37]. We showed that resveratrol protected BAEC migration from inflammatory conditions. However, the cell specific responses and molecular mechanisms require further investigation to fully resolve the contradictory observations of effects of resveratrol on EC migration.

Inflammation is defined in part by the upregulation of cell adhesion molecules on the surface of in response to cytokines. Adhesion molecule expression is the molecular basis of leukocyte-endothelium interactions and an important characteristic of inflammatory reactions, which are critical in atherogenesis [30]. We investigated the regulation of adhesion molecules by resveratrol with and without TNF-*α* in BAECs. We demonstrated for the first time that resveratrol inhibits TNF-*α*-induced expression of ICAM-1 and VCAM-1 during long-term incubation after TNF-*α* stimulation, as well as by pretreatment and cotreatment. There was no inhibitory effect of resveratrol on CAM expression during short-term incubation, and this characteristic may contribute to its time-dependent inhibition of NF-*κ*B [10]. E-selectin, the earliest adhesion molecule upregulated during leukocyte recruitment, was markedly increased after 4-h stimulation. We observed no significant inhibitory effects of resveratrol on the expression of *E*-selectin, which may be restricted by its long-term incubation. 

Resveratrol decreased TNF-*α*-induced ICAM-1 and VCAM-1 expression, which may protect cells from TNF-*α*-induced cytotoxicity. However, the underlying molecular mechanisms by which resveratrol exerts its physiological effects require further investigation. Together, our findings highlight the power of resveratrol to protect primary ECs in culture and suggest that resveratrol may offer an alternative therapy for the prevention and treatment of cardiovascular disease. Preclinical trials with baboons are planned to evaluate the protective effects of resveratrol.

## 5. Conclusions

In summary, our data suggest that resveratrol may protect baboon ECs from cytotoxicity induced by TNF-*α*. Resveratrol may provide a pharmacological approach for suppressing injury under inflammatory conditions and for reducing risk of cardiovascular disease and diabetes.
